# It Is Not Pneumocystis jiroveci (PCP), It Is Cyclophosphamide-Induced Pneumonitis

**DOI:** 10.7759/cureus.35263

**Published:** 2023-02-21

**Authors:** Mery Bartl, Yilen K Ng-Wong, Blesset Alexander, Jose G Gomez Casanovas, Josenny L Rodriguez- Paez, Andres Suarez, Christine E Loftis

**Affiliations:** 1 Internal Medicine, University of Texas Rio Grande Valley School of Medicine, McAllen, USA; 2 Internal Medicine, University of Texas Rio Grande Valley, McAllen, USA; 3 Internal Medicine, University of Texas Rio Grande Valley - DHR (Doctors Hospital at Renaissance), McAllen, USA

**Keywords:** cyclophosphamide, panca/mpo (myeloperoxidase)-positive microscopic polyangiitis (mpa), anca-associated vasculitis, ground-glass opacity, non-specific interstitial pneumonia, pauci-immune glomerulonephritis (gn), hispanic female, hispanic population, drug induced pneumonitis, anca associated vasculitis

## Abstract

Cyclophosphamide (CYC) is an immunosuppressive medication used to treat life-threatening complications of various rheumatic diseases like vasculitis and systemic lupus erythematosus. A rare side effect of this medication is pneumonitis, which occurs in less than 1% of patients. We describe a case of an 83-year-old woman with a past medical history of microscopic polyangiitis, who presented with progressive dyspnea at rest, exacerbated on exertion, and associated with orthopnea that was attributed to CYC-induced pneumonitis. Three months before this presentation, the patient was diagnosed with antineutrophil cytoplasmic antibodies (ANCA)-positive pauci-immune crescentic and necrotizing glomerulonephritis and started on CYC. On admission, a computed tomography (CT) chest showed worsening bilateral ground-glass opacities in a mosaic distribution and inter and intralobular septal thickening, not present on the CT performed three months prior. The patient underwent an extensive workup, which included an echocardiogram, bronchoscopy with bronchoalveolar lavage, and viral respiratory panel to rule out infectious and cardiac pathologies. She was started on empiric treatment with antibiotics and diuretics, however, despite these interventions, she continued with respiratory distress. A multidisciplinary team convened, and the diagnosis of CYC-induced lung injury was entertained. The CYC was discontinued, and the patient was started on prednisone with significant improvement in symptoms. This case highlights the importance of recognizing CYC as a rare cause of interstitial pneumonitis. When considering CYC-induced lung toxicity, other etiologies, such as opportunistic infections, cardiac etiologies, and diffuse alveolar hemorrhage, should be ruled out.

## Introduction

Cyclophosphamide (CYC) is an immunosuppressive medication used to treat life-threatening complications of various rheumatic diseases such as vasculitis and systemic lupus erythematosus [1]. Despite its rarity, it's important to perform serial pulmonary function tests in patients being treated with CYC [2]. When considering CYC-induced lung toxicity, other etiologies such as opportunistic infections and diffuse alveolar hemorrhage should be ruled out. We present a case of a patient with underlying microscopic polyangiitis (MPA) who presented with worsening hypoxemia and respiratory failure in the background of CYC therapy. This case highlights the importance of recognizing drug-related toxicities in our patients [3].

## Case presentation

**Figure 1:**
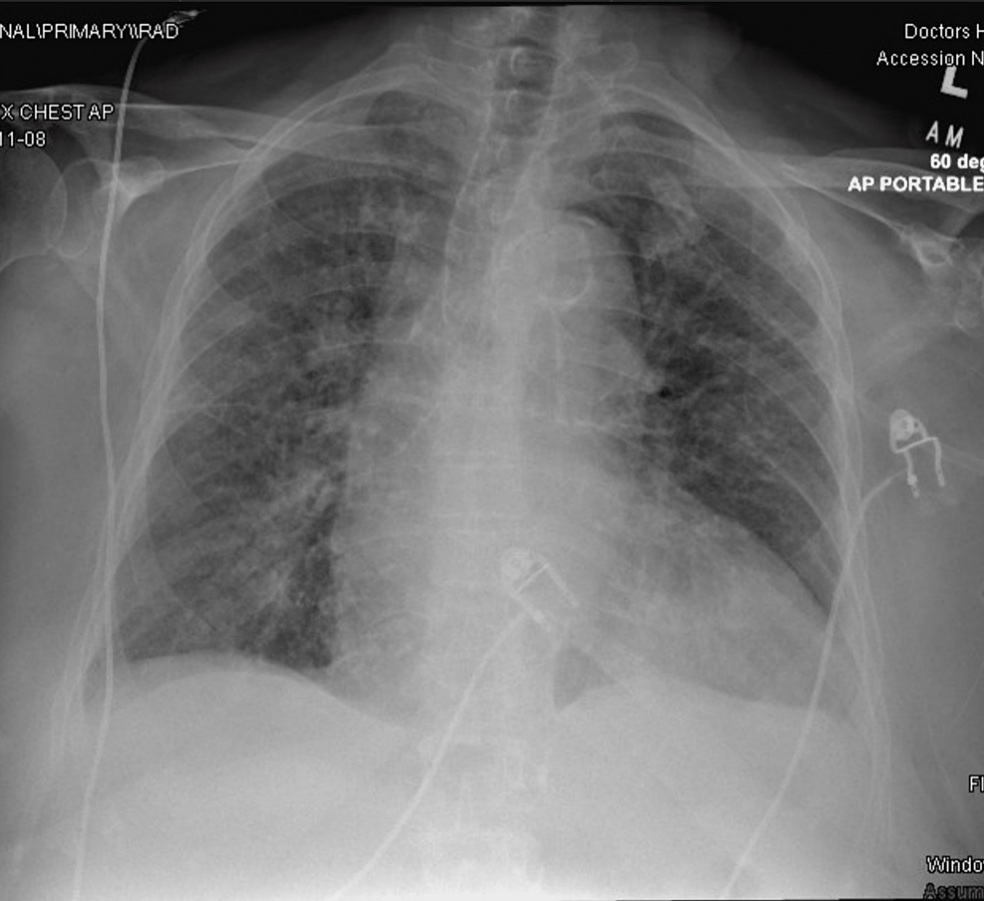
Chest X-ray showing increased interstitial markings suggestive of either atypical pneumonia versus pulmonary edema versus interstitial pneumonitis

The patient is an 83-year-old woman with a past medical history of microscopic polyangiitis (MPA), hypertension, and hypothyroidism who presented to the emergency department due to progressive dyspnea at rest exacerbated on exertion, associated with orthopnea. The patient was admitted due to acute-on-chronic hypoxemic respiratory failure with multifactorial etiology and heart failure exacerbation with a preserved ejection fraction of 65%. Three months before this admission, the patient was diagnosed with antineutrophil cytoplasmic antibodies (ANCA)-positive pauci-immune crescentic and necrotizing glomerulonephritis; she was started on cyclophosphamide 75 mg in the morning and 100 mg at night, as well as prednisone 10 mg daily. On admission, vitals were BP 176/76 mmHg, HR 78 bpm, RR 20 breaths per minute, with oxygen saturation of 96% on 5 L of nasal cannula. The physical examination revealed a grade III systolic murmur at the right upper parasternal border and inspiratory crackles on both lung bases. Laboratory studies were remarkable for pancytopenia, with normocytic anemia with a hemoglobin of 9.3 cells/uL, a white blood cell (WBC) count of 3.20 cells/uL, a hemoglobin of 7.9 gm/dl, a hematocrit of 22.3 %, a platelet count of 117 cells/uL, and a brain natriuretic peptide (BNP) of 62 pg/mL. A chest X-ray was performed and showed increased interstitial markings suggestive of either atypical pneumonia versus pulmonary edema versus interstitial pneumonitis (Figure 1).

**Figure 2:**
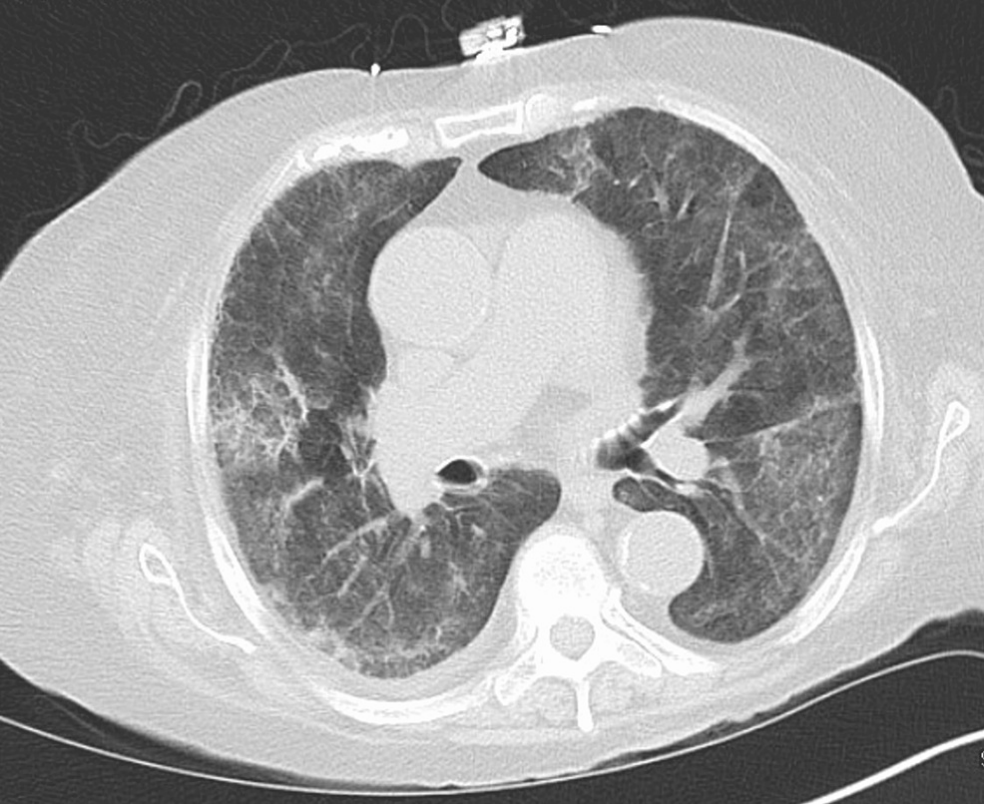
Follow-up CT chest without contrast showing worsening bilateral ground-glass densities in a mosaic distribution and inter and intralobular septal thickening CT: computed tomography

**Figure 3:**
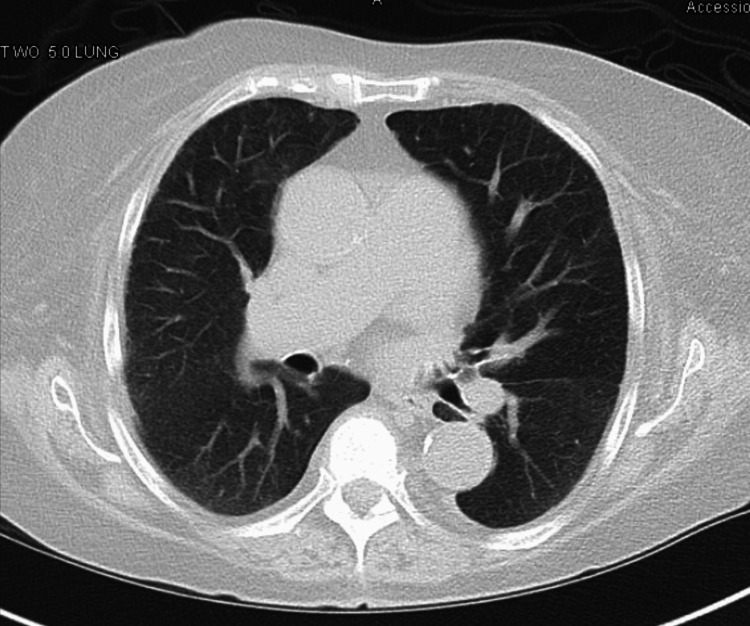
CT chest without contrast showing very mild scattered ground-glass opacities CT: computed tomography

The patient was started on empiric coverage for opportunistic infections with clindamycin, primaquine, azithromycin, and diuretics; however, the patient’s symptoms did not improve nor was there any radiographic improvement. Subsequently, the patient underwent an echocardiogram, which revealed moderate aortic stenosis, normal systolic function with an ejection fraction of 66%, and a right systolic ventricular pressure of 65 mmHg suggestive of pulmonary hypertension-likely Group 2; however, this could not explain the patient’s sudden onset of the development of symptoms. A multidisciplinary group, including an infectologist, cardiologist, pulmonologist, and nephrologist evaluated the patient for other plausible etiologies. CT chest without contrast demonstrated worsening bilateral ground-glass densities in a mosaic distribution and inter and intralobular septal thickening (Figure 2), which were not present on previous CT performed three months prior (Figure 3).

Bronchoscopy ruled out diffuse alveolar hemorrhage, and bronchoalveolar lavage (BAL) was negative for fungal or mycobacterium. Grocott methenamine silver (GMS) stain negative for acid-fast bacilli, Pneumocystis jiroveci (PCP), and Aspergillus. The viral respiratory panel also was negative. Having ruled out other etiologies, a consensus was made that the patient’s symptoms were due to interstitial pneumonitis secondary to CYC that was started three months prior. Alveolar hemorrhage was ruled out as BAL came negative, and the CT scan was not compatible with the usual pulmonary involvement in MPA. Antibiotics were de-escalated, and the patient was discharged home on oxygen. A decision was made to discontinue the patient's CYC, and she was started on a prednisone taper with close follow-up. Renal function remained stable afterward.

## Discussion

CYC is an alkylating agent well-established in the treatment of ANCA vasculitis, with the most common side effects being related to myelosuppression. Rarely, CYC can cause lung toxicity; however, this occurs in less than 1% of the population [4]. The recognition and diagnosis of CYC-induced lung injury may be difficult because of the presence of confounding variables such as concomitant use of other cytotoxic drugs, opportunistic infections, diffuse pulmonary malignancy, radiation pneumonitis, and oxygen toxicity [4-6]. The mechanism of toxicity is not well understood, however, the literature reports that the most likely causative metabolite is called acrolein [7]. Although there are only a few reported cases, two types of pneumonitis caused by CYC have been described: early-onset type and late-onset type. The early onset pneumonitis is often reversible and the most common symptoms are cough and dyspnea within six months of receiving CYC therapy. The treatment of early-onset CYC-induced pneumonitis consists of medication discontinuation and corticosteroid administration. On the contrary, the late onset of pneumonitis is irreversible, usually not responsive to corticosteroids, and confers a worse prognosis.

Histopathology of lung tissue from patients with CYC-induced lung toxicity shows a mixture of inflammatory cells, activated fibroblasts, abnormal alveolar cells, and collagen fibers [8]. Radiographically, bilateral pleural thickening is commonly present; however, scattered or diffuse areas of ground-glass opacities, interlobular septal thickening, fibrosis, or consolidation have been described [9]. A review of patients treated with CYC at the Mayo Clinic between 1974 and 1994 was only able to confirm six cases of CYC-induced lung toxicity while other causes of lung injury could not be excluded in an additional 29 patients. Of these patients, five were men and the age of onset ranged between 42 and 78 years [6]. Out of these six patients, only one presented with early-onset pneumonitis that responded to the discontinuation of the offending drug. Patients with late-onset pneumonitis showed no response to the cessation of cyclophosphamide and initiation of corticosteroid therapy. Moreover, Taywade et al. described drug-induced pulmonary toxicity in two patients as a side effect of neoadjuvant treatment with CYC for breast cancer, and one of the patients recovered upon discontinuation of therapy [10].

Our patient had multiple etiologies that could account for her shortness of breath, including moderate aortic stenosis and pulmonary hypertension. However, she was recently started on CYC, and the CT scan of the chest revealed worsening ground-glass opacities and pleural thickening, which were not present on her previous hospital admission. Infectious etiology was in the differential, however, further studies ruled out infectious causes. Interstitial lung disease in the usual interstitial pneumonia pattern has been described as the most common pulmonary involvement as a sequela of MPA [11]. Radiographic features of ANCA-associated vasculitis with interstitial lung disease include ground-glass opacities, interlobular septal thickening, honeycombing, reticular patterns, and traction bronchiectasis [12]. Interestingly, early-stage CYC-induced pneumonitis is characterized radiographically with peripheral ground glass opacities, which correlated with the findings in our patient, and although it is described in diffuse alveolar hemorrhage, BAL came back negative. Given our patient’s clinical presentation, recent initiation of CYC, no concomitant medications that could have explained symptomatology, and radiographic findings, the diagnosis of CYC-induced pneumonitis was the most plausible. Our patient’s symptoms improved after the discontinuation of CYC and low-dose prednisone, which is consistent with the response seen in early-onset CYC-induced pneumonitis. Renal function has remained stable.

## Conclusions

In this case, the patient presented to the emergency department with progressive shortness of breath and respiratory failure. After all other causes were ruled out, CYC-induced pneumonitis was diagnosed. The patient's symptoms started within six months of initiation therapy for ANCA vasculitis, which placed the patient in the early-onset type, which confers a more favorable outcome. The CYC was discontinued, and corticosteroids were initiated. Although there are several causes of worsening ground glass opacities in patients with ANCA vasculitis, clinical history and diagnostic tests are imperative to narrow the differential. Pulmonary toxicity associated with CYC is often underscored by the low prevalence rates in patients with ANCA-associated vasculitis, so it is essential to have a high index of suspicion when considering this in the differential.
